# Identification of differentially expressed miRNAs and mRNAs in synovial of osteoarthritis via RNA-sequencing

**DOI:** 10.1186/s12881-020-0978-5

**Published:** 2020-03-02

**Authors:** Yu Zhou, Zhicong Wang, Xi Chen, Jianjun Zhang, Ling Yang, Shuping Liu, Yuehong Liu

**Affiliations:** Department of Orthopedics, People’s Hospital of Deyang City, No. 173, Taishan North Road, Jingyang District, Deyang, 618000 Sichuan China

**Keywords:** Osteoarthritis (OA), mRNA, miRNA, RNA-sequencing, Functional annotation

## Abstract

**Background:**

Osteoarthritis (OA) is the most common form of arthritis and a leading cause of disability. This study attempted to investigate the key mRNAs and miRNAs related to OA.

**Patients and methods:**

From April 17th, 2018 to May 17th, 2018, five patients with OA and three normal controls were enrolled in this present study. To identify the differentially expressed mRNAs (DEmRNAs) and miRNAs (DEmiRNAs) between patients with OA and normal controls, RNA-sequencing was performed. Then, DEmiRNA-target DEmRNAs analysis and functional annotation of DEmiRNA-target DEmRNAs were performed. To validate the RNA-sequencing results, quantitative real time-PCR (RT-PCR) and western blot analysis were performed as well.

**Results:**

A total of 1068 DEmRNAs, 21 DEmiRNAs and 395 DEmiRNA-DEmRNA pairs were identified in synovial tissues of patients with OA. The functional annotation of DEmiRNA-target DEmRNAs revealed that Pathways in cancer and PI3K-Akt signaling pathway were significantly enriched Kyoto Encyclopedia of Genes and Genomes (KEGG) pathways. QRT-PCR and western blot results revealed that except for TLR7, the expression level of the others was consistent with the RNA-sequencing results, generally.

**Conclusion:**

The findings of this present study may provide new clues for the roles of DEmRNAs and DEmiRNAs in the pathogenesis of OA.

## Introduction

Osteoarthritis (OA) is the most frequent musculoskeletal disease and leads to functional decline and loss in quality of life [1]. Currently, there is no effective treatment to prevent the initiation and progression of the disease while the severity of OA often worsens with age [2]. Reportedly, it estimates that among population over 60 years old, there was 9.6% of men and 18% of women suffer from symptomatic OA in the worldwide [3]. Although the disease is with a trait of destruction of articular cartilage, pathological changes in subchondral bone and associated synovitis, the pathogenesis is poor understanding which is thought to be multifactorial [4]. Inability to diagnose early and poorly understanding of the pathophysiology makes early diagnosis to be a key factor in the prevention and management of disease [5]. Recently, molecular biology has been reported to play a vital role in explaining its disease pathophysiology, and gene regulation has been demonstrated to be involved in driving an imbalance between the expression of catabolic and anabolic factors, leading eventually to osteoarthritic cartilage degeneration [6]. There is increasing attention on the influence of dysregulation at a molecular level on the pathogenic process.

MicroRNAs (miRNAs) have been detected widely in eukaryotes to play roles in regulating growth, development, differentiation, and metabolism in model organisms which are short (approximately 22 nucleotides), non-coding, RNA regulators of gene expression [7]. Little was known of the function of miRNAs at the stage when it is first identified in the early 1990’s by Lee et al. (in *Caenorhabditis elegans*) [8]. Due to the link between alterations in miRNA expression levels and multifarious disease processes have been linked, aberrant expression in pathological states has drawn public attention [9]. Studies based on highly specific patterns of miRNA expression correlate with development and several diseases have revealed the potential for therapeutic manipulation of miRNAs [10, 11]. Recently, a large number of studies have been done to explore miRNAs and genes associated with OA [5, 6, 12–14]. Even so, studies on biomarkers for OA are still urgent to be performed.

In this study, differentially expressed miRNAs (DEmiRNA) and mRNAs (DEmRNAs) in synovial tissues of patients with OA were identified by RNA-sequencing. DEmiRNA-target DEmRNAs analysis and functional annotation of DEmiRNA-target DEmRNAs were performed. Quantitative real time-PCR (RT-PCR) and western blot analysis were performed to validate the RNA-sequencing results. In doing so, we hope this study could represent a new avenue to understand the pathogenesis and develop potential biomarkers for OA.

## Materials and methods

### Patients and samples

Five patients with OA and three normal controls were recruited in this study from April 17th, 2018 to May 17th, 2018. According to the criteria of the American College of Rheumatology, OA were diagnosed [15]. Healthy employees with no symptoms or signs of OA, or any other type of arthritis, or any painful condition of the joints, were included as controls. Beside, participants with no personal or family history of OA were selected as control subjects. The participants with history of joint diseases, including inflammatory arthritis (rheumatoid arthritis or any other autoimmune disease), post-traumatic or post-septic arthritis, poliomyelitis, skeletal dysplasia, were excluded. Radiographic evaluation of all participants were performed. Table 1 displayed the detailed information of all these participants. The written informed consent for use of their samples from every participant were provided in the present study. Ethical approval for this study was granted by the ethics committee of People’s Hospital of Deyang City (2017–043). Synovial tissues of every participant were obtained.

**Table 1:** Patient characteristics

Index	Weight (kg)	Height (cm)	Kellgren Lawrence grade (n)	Disease duration	Blood glucose (mmol/L)	CRP (mg/L)	ESR (mm/h)	Part
Case 1	56–75	136–165	4	8 years	5.63	3.44	48	Left knee
Case 2			1	2 months	5.98	2.9	8	Left knee
Case 3			4	30 years	5.08		14	Right knee
Case 4			2	10 years	6.2	27.94	16	Left knee
Case 5			4	4 years	5.1	0	16	Right knee
Control 1	46–60	155–165	–	3 h		–	–	Right knee
Control 2			–	2 h	4.62	–	–	Right knee
Control 3			–	1 day	5.2	–	–	Left knee

*CRP* C-reactive protein, *ESR* Erythrocyte sedimentaition ratio

### RNA isolation and sequencing

Following the manufacturer’s protocol, we used TRIzol reagent (Invitrogen, Carlsbad, CA, USA) to isolate total RNA from samples. The concentration and purity of RNA was determined with Nanodrop ND-2000 spectrophotometer (Thermo Fisher Scientific, Wilmington, DE, USA), and the integrity of RNA was confirmed via a 2% agarose gel. With an Agilent 2100 Bioanalyzer (Agilent, Palo Alto, CA, USA), the RNA integrity number (RIN) value was obtained. With QiaQuick PCR Purification Kit, the mRNA library was constructed. The 18–30 nt RNA was obtained from the total RNA. By using TruseqTM Small RNA Sample Prep Kit, adapter ligation and reverse transcription polymerase chain reaction (PCR) were performed to obtain the cDNA. Sequencing was performed based on HiSeq x-ten platform (Illumina) and SE50, BGIseq, respectively.

### Identification of DEmRNAs in patients with OA compared with normal controls

The clean reads were aligned with the human reference genome, Ensemble GRCh38.p7 (ftp://ftp.ncbi.nlm.nih.gov/genomes/Homo_sapiens) by using TopHat release 2.2.1 (http://tophat.cbcb.umd.edu/). With Cuffquant version 2.2.1 (http://cufflinks.cbcb.umd.edu/), expression of mRNAs was normalized and outputted. To determine the transcription abundance of mRNAs, fragments per Kilobase of exon per million fragments mapped (FPKM) was used. With Cuffdiff version 2.2.1 (http://cufflinks.cbcb.umd.edu/), FPKMs of mRNAs were calculated. Differentially expressed mRNAs (DEmRNAs) were identified with *p* < 0.05 and |log_2_FC| > 1. Hierarchical clustering analysis of DEmRNAs was conducted with R package “pheatmap”. The flow chart of the analyses was showed in Figure S1.

### Functional annotation of DEmRNAs between patients with OA and normal controls

To further research the biological function of DEmRNAs, Gene Ontology (GO) function and Kyoto Encyclopedia of Genes and Genomes (KEGG) pathway enrichment analyses of the DEmRNAs between patients with OA and normal controls were performed by DAVID 6.8 (https://david.ncifcrf.gov/). A value of *p* < 0.05 was defined as the criteria of statistical significance.

### Identification of DEmiRNAs in patients with OA compared with normal controls

With Bowtie (bowtie-bio.sourceforge.net), the alignment between the cleaned miRNA sequencing reads was aligned to the human genome (GRCh38.p7 assembly) based on Genome human UCSC reference annotation. With miRDeep2 (https://www.mdc-berlin.de/8551903/en/), the transcription abundance of miRNAs was determined. Based on the read count of each sample, the DEmiRNAs in OA compared to normal controls were calculated with an R-bioconductor package, DESeq2 (http://www.bioconductor.org/packages/release/bioc/html/DESeq2.html). For DEmiRNAs in OA compared to normal controls, the threshold was defined as base Mean > 100, *p* < 0.05 and |log_2_FC| > 2. With R package “pheatmap”, hierarchical clustering analysis of DEmRNAs was conducted.

### DEmiRNA-target DEmRNAs analysis

Given miRNAs tend to decrease the expression of their target mRNAs, we selected target genes from DEmRNAs that expressed inversely with that of miRNA for further research. Firstly, the putative targeted DEmRNAs of DEmiRNAs were predicted by six bioinformatic algorithms (RNA22, miRanda, miRDB, miRWalk, PICTAR2 and Targetscan). Then, with miRWalk, the confirmed targeted DEmRNAs of DEmiRNAs were obtained. Thirdly, the confirmed DEmiRNA-DEmRNA pairs were derived from miRWalk and the DEmiRNA-DEmRNA pairs recorded by ≥4 algorithms. Based on the obtained DEmiRNA-DEmRNA pairs, DEmiRNA-DEmRNA interaction networks between OA and normal controls were constructed by using Cytoscape software (http://www.cytoscape.org/).

### Functional annotation of DEmiRNA targets

To further research the biological function of the target DEmRNAs of DEmiRNAs, GO analysis was performed t by using Gorilla (http://cbl-gorilla.cs.technion.ac.il/) with a *p*-value < 0.01, and KEGG pathway analysis was performed by using webgestalt (http://www.webgestalt.org/) with a *p*-value < 0.05.

### Quantitative real time-PCR (RT-PCR) and western blot analysis

Eight synovial tissues were obtained from four patients with OA and four normal controls. The written informed consent for use of their samples from every participant were provided in the present study. Ethical approval for this study was granted by the ethics committee of People’s Hospital of Deyang City (2017–043).

Total RNA was isolated with the Trizol reagent (Invitrogen, USA). The qRT-PCR reactions were performed based on SuperReal PreMix Plus (Invitrogen, USA) in ABI 7300 Real-time PCR Detection System. With 2^-ΔΔCt^ method, relative gene expression was determined. The human GAPDH and ACTB were used as endogenous controls for mRNA expression, and the human hsa-U6 was used as endogenous controls for miRNA expression in analysis.

Synovial tissues were lysed on ice with RIPA lysis buffer, and then the supernatants were collected by centrifugation at 12,000 rpm at 4 °C for 30 min. The protein concentrations were detected with BCA protein assay. The protein extracts were separated by 10% SDS-PAGE, transferred onto PVDF membranes, and probed using primary and then secondary antibodies. The primary antibodies were as follows: rabbit anti-GAPDH, TIMP3 Antibody, CTSS Antibody and TLR7 Antibody. The blots were visualized with ECL reagent.

## Results

### DEmRNAs and DEmiRNAs between patients with OA and normal controls

**Fig. 1:**
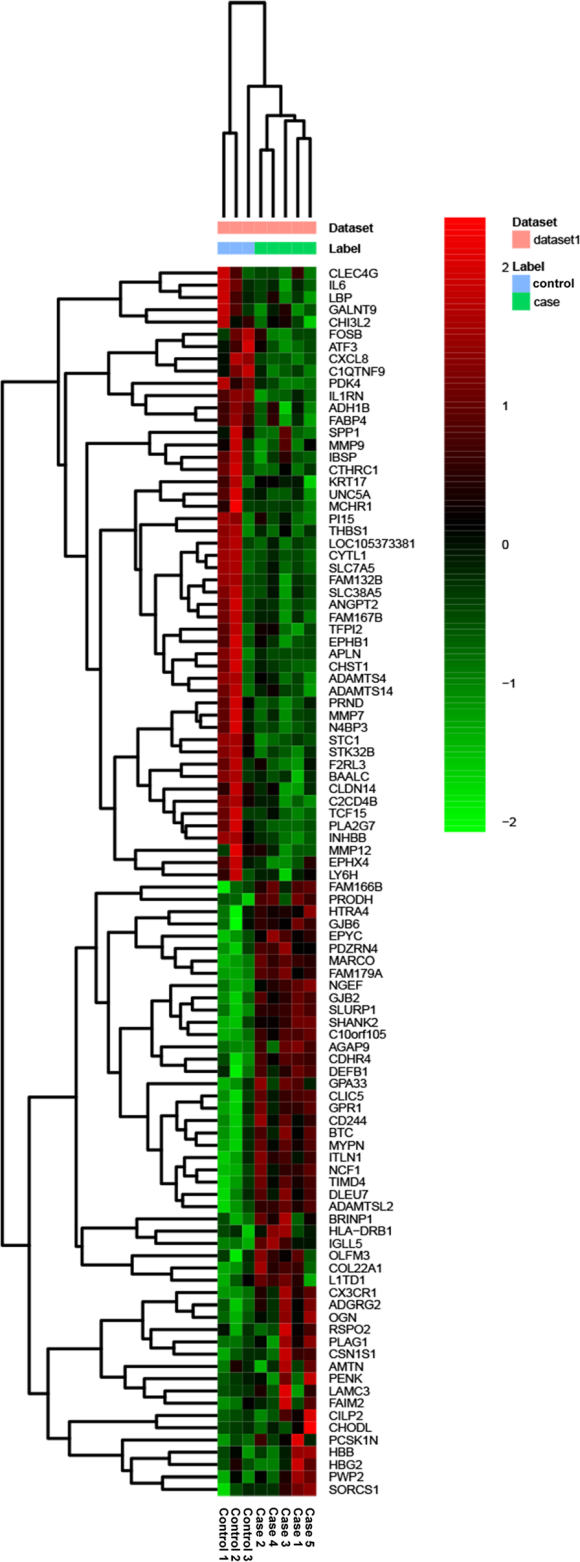
The heatmap of top 50 up- and down-regulated DEmRNAs between OA and normal controls. Row and column represented DEmRNAs and tissue samples, respectively. The color scale represented the expression levels. The red and green color represented the up- and down-regulated

**Fig. 2:**
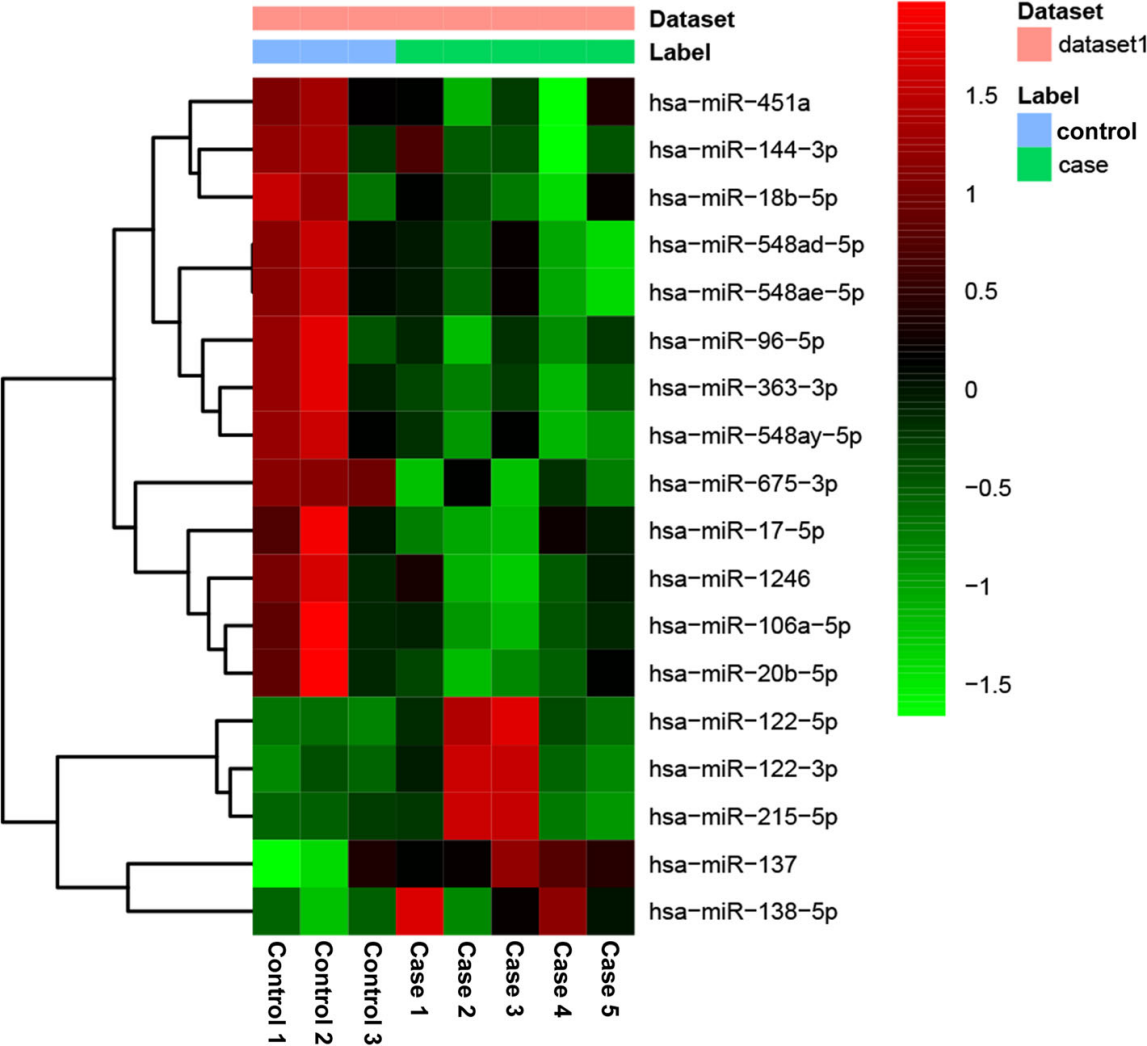
The heatmap of DEmiRNAs between OA and normal controls. Row and column represented DEmiRNAs and tissue samples, respectively. The color scale represented the expression levels. The red and green color represented the up- and down-regulated

Expression profiling of mRNA and miRNA extracted from five patients with OA and three normal controls identified 1068 DEmRNAs (516 up-regulated and 552 down-regulated DEmRNAs) and 21 DEmiRNAs (6 up-regulated and 15 down-regulated DEmiRNAs). The top 10 up- and down-regulated DEmRNAs and all DEmiRNAs were showed in Table 2 and Table 3, respectively. Hierarchical clustering analysis of top 50 up- and down-regulated DEmRNAs and DEmiRNAs was displayed in Fig. 1 and Fig. 2, respectively. The raw-data have been uploaded to Gene Expression Omnibus (GEO) (GSE143514, https://www.ncbi.nlm.nih.gov/geo/query/acc.cgi?acc=GSE143514).

**Table 2:** Top 10 up- and down-regulated DEmRNAs between patients with OA and normal controls

ID	Symbol	log_2_FC	*p*-value	Regulation
91,937	TIMD4	3.81464	5.00E-05	up
57,152	SLURP1	3.86063	0.0037	up
27,344	PCSK1N	3.93602	0.00065	up
55,600	ITLN1	4.00229	0.0016	up
25,791	NGEF	4.17476	5.00E-05	up
140,578	CHODL	4.2872	0.01285	up
100,423,062	IGLL5	4.79462	0.0298	up
1446	CSN1S1	5.81655	5.00E-05	up
91,937	TIMD4	3.81464	5.00E-05	up
57,152	SLURP1	3.86063	0.0037	up
4316	MMP7	−7.00868	0.00845	down
8862	APLN	−5.19773	5.00E-05	down
3381	IBSP	−4.29821	0.00195	down
54,360	CYTL1	−4.23704	5.00E-05	down
6781	STC1	−4.21422	5.00E-05	down
3569	IL6	−4.15517	5.00E-05	down
4321	MMP12	−4.03152	0.0094	down
90,249	UNC5A	−3.84362	0.03125	down
7980	TFPI2	−3.81017	0.0099	down
7941	PLA2G7	−3.78853	0.02245	down

*DEmRNAs* Differentially expressed mRNAs, *FC* Fold change

**Table 3:** DEmiRNAs between patients with OA and normal controls

miRNA|precursor	baseMean	log_2_FC	*p*-value	Regulation
hsa-miR-106a-5p|hsa-mir-106a	492.1284	−3.19961	0.000226	Down
hsa-miR-1246|hsa-mir-1246	258.1751	−2.0786	0.011492	Down
hsa-miR-144-3p|hsa-mir-144	17,728.54	−3.31783	0.049975	Down
hsa-miR-17-5p|hsa-mir-17	7597.817	−2.22172	0.004888	Down
hsa-miR-18b-5p|hsa-mir-18b	109.0147	−2.1827	0.012516	Down
hsa-miR-20b-5p|hsa-mir-20b	1693.174	−3.26326	0.000196	Down
hsa-miR-363-3p|hsa-mir-363	4384.713	−2.63676	0.000153	Down
hsa-miR-451a|hsa-mir-451a	5,294,654	−2.72244	0.005101	Down
hsa-miR-548ad-5p|hsa-mir-548ad	110.1592	−2.59423	0.000577	Down
hsa-miR-548ad-5p|hsa-mir-548ay	161.6666	−2.04144	0.001393	Down
hsa-miR-548ae-5p|hsa-mir-548ad	110.1592	−2.59423	0.000577	Down
hsa-miR-548ae-5p|hsa-mir-548ay	161.6666	−2.04144	0.001393	Down
hsa-miR-548ay-5p|hsa-mir-548ay	161.6666	−2.04144	0.001393	Down
hsa-miR-675-3p|hsa-mir-675	115.7026	−2.11926	0.001639	Down
hsa-miR-96-5p|hsa-mir-96	2277.136	−3.01869	0.000203	Down
hsa-miR-122-3p|hsa-mir-122	182.4923	8.118732	0.000981	Up
hsa-miR-122-5p|hsa-mir-122	2707.5	6.661302	2.67E-05	Up
hsa-miR-137|hsa-mir-137	351.0572	2.317024	0.002847	Up
hsa-miR-138-5p|hsa-mir-138-1	1356.231	2.898852	0.000224	Up
hsa-miR-138-5p|hsa-mir-138-2	307.7424	2.781289	0.000927	Up
hsa-miR-215-5p|hsa-mir-215	518.2554	5.316112	0.001199	Up

*DEmiRNAs* Differentially expressed miRNAs, *FC* Fold change

### Functional annotation of DEmRNAs between patients with OA and normal controls

**Fig. 3:**
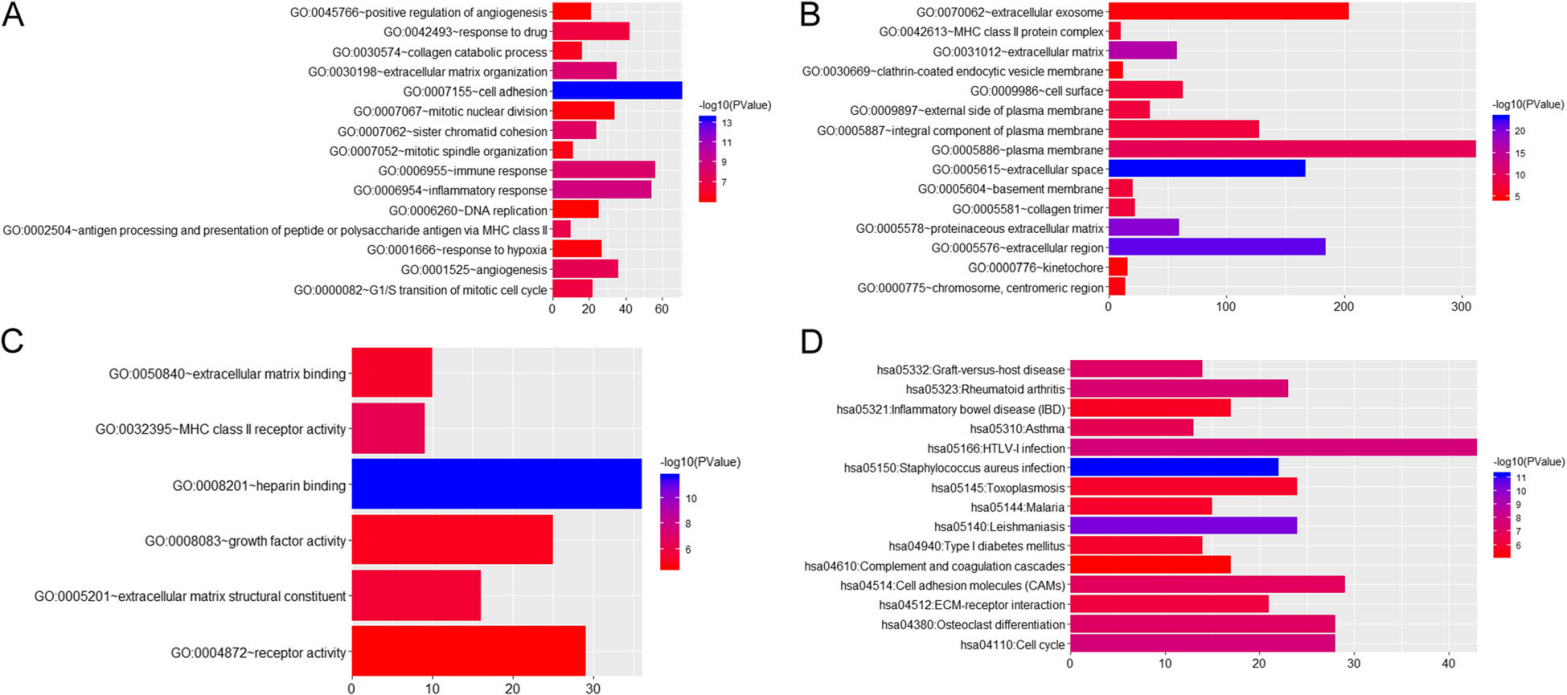
Significantly enriched GO terms and KEGG pathways of DEmRNAs between OA and normal controls. **a**. BP, biological process; **b**. CC, cellular component; **c**. MF, molecular function; **d** KEGG pathways. The x-axis shows counts of DEmRNAs enriched in GO terms or KEGG pathways and the y-axis shows GO terms or KEGG pathways. The color scale represented -lg *p*-value

Protein phosphorylation (*p* = 1.84E-05), defense response to virus (*p* = 3.54E-05), positive regulation of transcription from RNA polymerase II promoter (*p* = 4.08E-05), extracellular space (*p* = 7.09E-24) and heparin binding (*p* = 2.03E-12) were significantly enriched GO terms in OA (Fig. 3a-c). *Staphylococcus aureus* infection (*p* = 5.90E-12), Leishmaniasis (*p* = 4.66E-11) and HTLV-I infection (*p* = 1.84E-08) were significantly enriched KEGG pathways in OA (Fig. 3d).

### DEmiRNA-target interactions

**Fig. 4:**
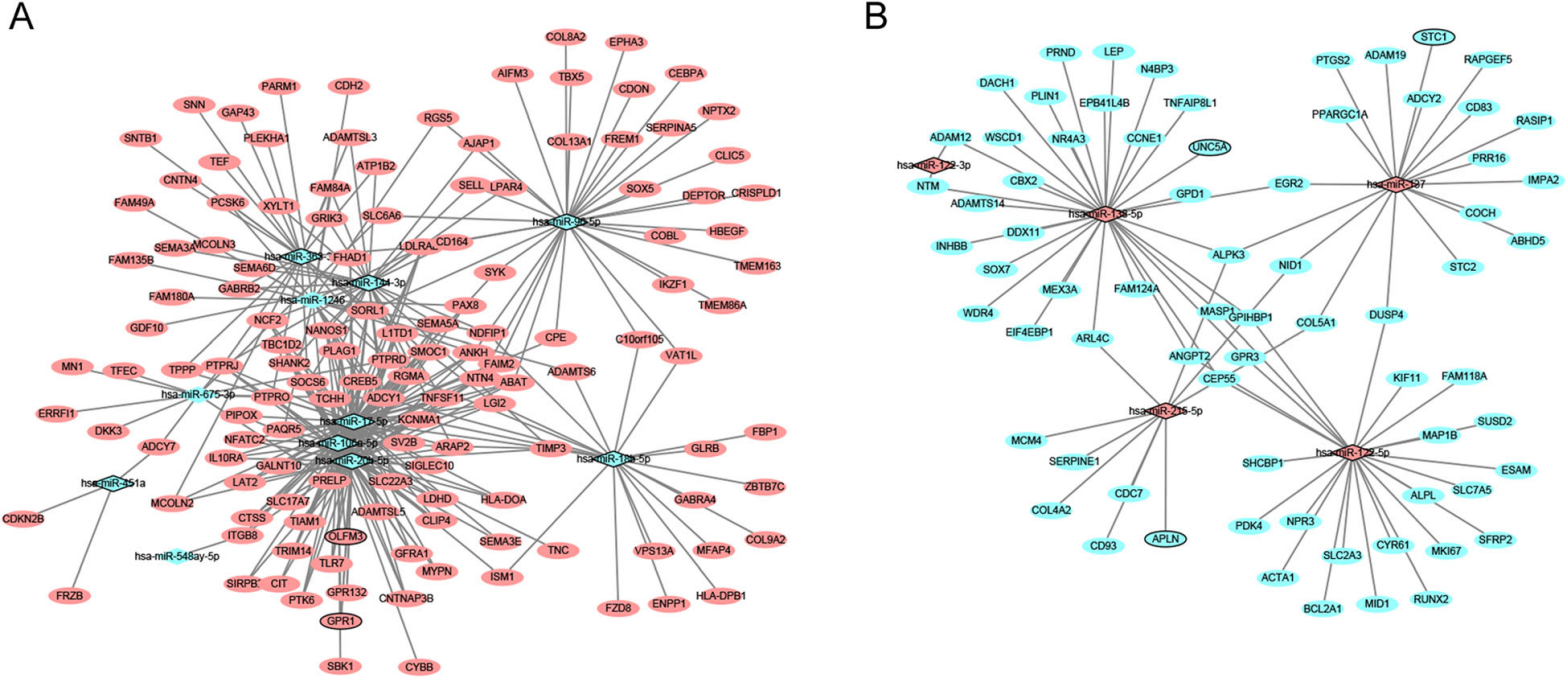
DEmiRNA-DEmRNA interaction network. **a** Interaction network between down-regulated DEmiRNAs and up-regulated DEmRNAs; **b** Interaction network between up-regulated DEmiRNAs and down-regulated DEmRNAs. The rhombic nodes and elliptical nodes indicate DEmiRNAs and DEmRNAs, respectively. Red and green color represent up-regulation and down-regulation, respectively.

A total of 395 DEmiRNA-DEmRNA pairs, including 376 DEmiRNA-DEmRNA pairs which were predicted by ≥4 algorithms and 48 validated DEmiRNA-DEmRNA pairs derived from the miRWalk, were obtained (Fig. 4). Among which, hsa-miR-17-5p (degree = 62), hsa-miR-20b-5p (degree = 56) and hsa-miR-106a-5p (degree = 52) were the top three DEmiRNAs that covered most DEmRNAs.

### Functional annotation of DEmiRNA targets

**Fig. 5:**
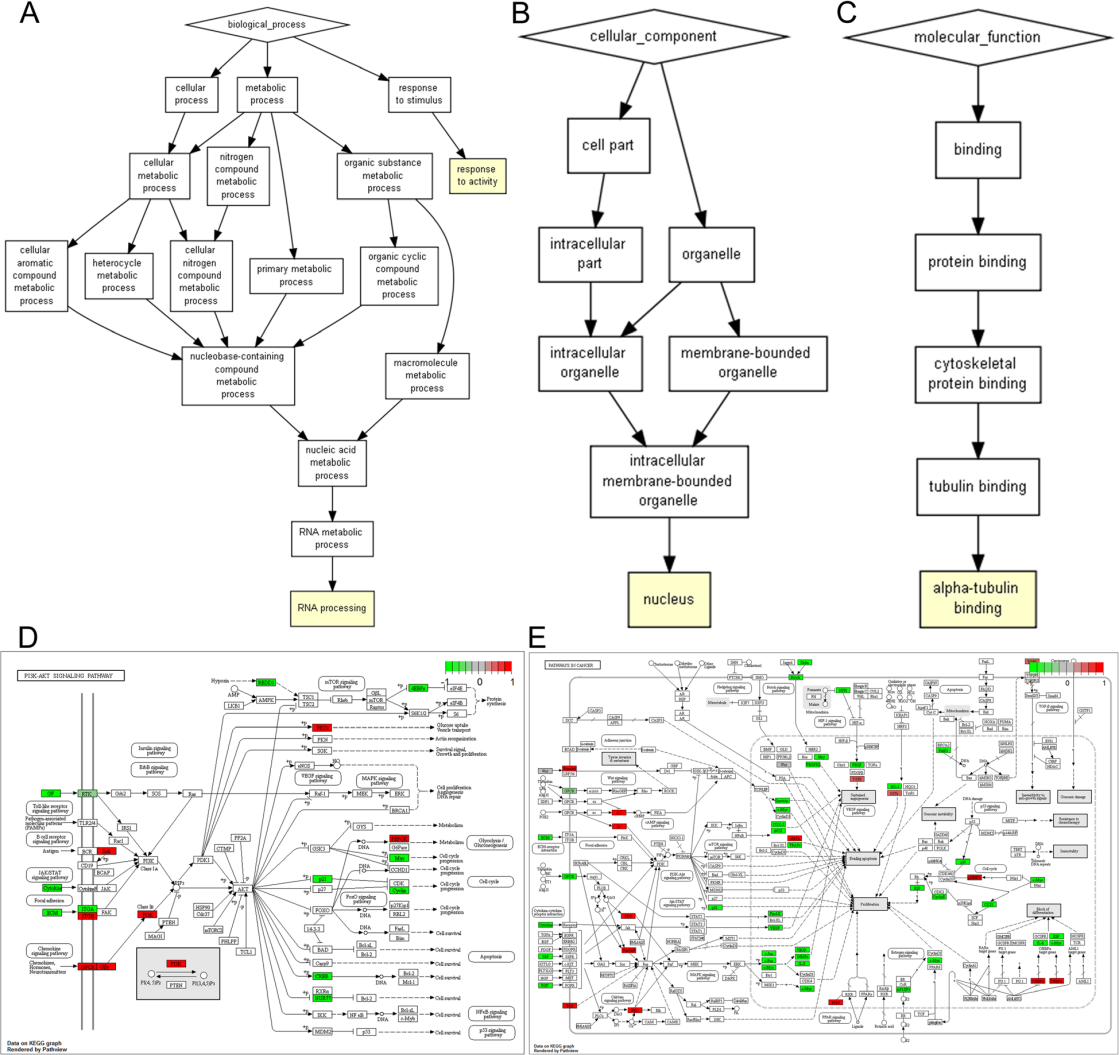
Significantly enriched GO terms and KEGG pathways of DEmiRNA-target DEmRNAs. **a**. BP, biological process; **b**. CC, cellular component; **c**. MF, molecular function. **d-e** KEGG pathways. **d**. PI3K-Akt signaling pathway. **e**. Pathways in cancer. The red and green rectangles represented the particles which regulated by the up- and down-regulated target DEmRNAs of DEmiRNAs, respectively. Appropriate copyright permission to use the signalling pathways was obtained

Base on GO enrichment analysis, RNA processing (*p* = 5.80E-04), response to activity (*p* = 9.04E-04), nucleus (*p* = 3.56E-04) and alpha-tubulin binding (*p* = 5.80E-04) were significantly enriched GO terms in OA (Fig. 5a-c). According to the KEGG pathway enrichment analysis, the DEmiRNA-target DEmRNAs were significantly enriched in Pathways in cancer (*p* = 3.36E-02) and PI3K-Akt signaling pathway (*p* = 4.67E-02) (Fig. 5d-e) [16].

### The results of qRT-PCR and western blot

**Fig. 6:**
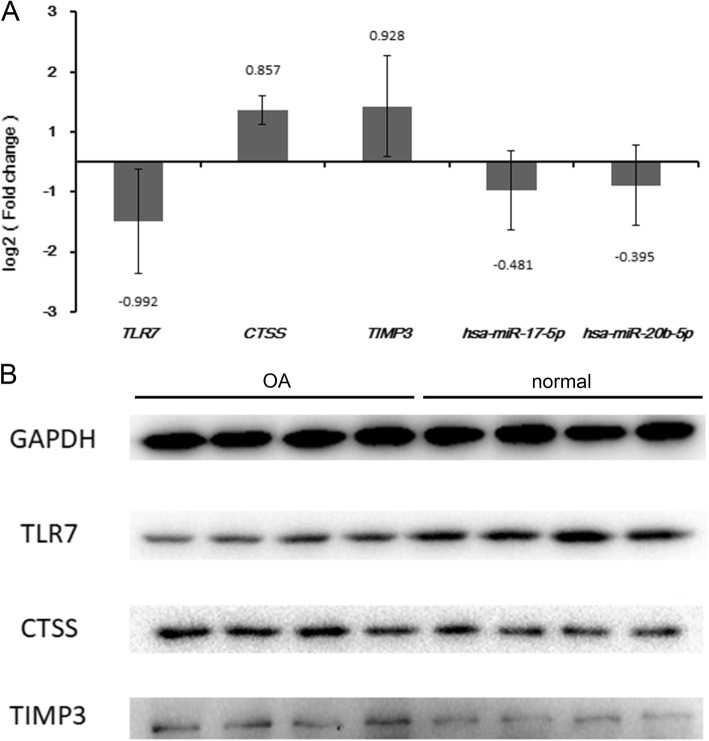
The results of qRT-PCR and western blot. **a**. The quantitative real time polymerase chain reaction (qRT-PCR) results of the DEmRNAs and DEmiRNAs in OA. The x-axis represents the DEmRNAs/DEmiRNAs and the y-axis represents log_2_ (fold change). **b**. Western blotting analysis of TLR7, CTSS and TIMP3 protein levels in OA and normal controls. GAPDH protein was used as the loading control

Three DEmRNAs (including TLR7, CTSS and TIMP3) and two DEmiRNAs (including hsa-miR-17-5p and hsa-miR-20b-5p) were selected to use for qRT-PCR validation. Based on the results of RNA sequencing, TLR7, CTSS and TIMP3 were up-regulated while hsa-miR-17-5p and hsa-miR-20b-5p were down-regulated in OA. Except for TLR7, expression of the others in the qRT-PCR results was consistent with that in this present study, generally (Fig. 6a). Western blot results revealed that the protein level of CTSS and TIMP3 were up-regulated while the protein level of TLR7 was down-regulated in OA (Fig. 6b).

## Discussion

OA is a progressive disease with a long silent period, which shows signs of cartilage degradation, mild-to-moderate synovial inflammation and altered bone structure, resulting in severe destruction and impaired function of the affected joints [17]. This study performed RNA-sequencing to attempt to obtain the key miRNAs and mRNAs associated with OA. Here, we discussed two DEmiRNAs (including hsa-miR-17-5p and hsa-miR-20b-5p), which were the top two DEmiRNAs that covered most DEmRNAs, and three DEmRNAs (including TLR7, CTSS and TIMP3), which were target genes of the two DEmiRNAs mentioned above.

Toll like receptor 7 (TLR7), a member of the Toll-like receptor (TLR) family, encoded by TLR7, plays a fundamental role in pathogen recognition and activation of innate immunity [18]. From Drosophila to humans, TLRs are highly conserved and share structural and functional similarities [18]. In humans, the TLR family, a set of 10 type I transmembrane receptors, have specificity for different types of pathogen-associated molecular patterns (PAMPs) [19]. PAMPs are expressed on infectious agents, and mediate the production of cytokines necessary for the development of effective immunity [19]. In humans, ten TLRs have been identified, and TLR-1, TLR-2, TLR-4, TLR-5, TLR-6, and TLR-10 are expressed on the cell surface while TLR-3, TLR-7, TLR-8, and TLR-9 are expressed in endosomes [20]. TLR-7 recognizes single-stranded RNA (ssRNA) in endosomes and is activated by the synthetic antiviral compound imiquimod [21]. TLR activation has been demonstrated to be involved in the development of synovitis, degeneration of cartilage, and susceptibility to disease in OA [22]. Chamberlain et al. suggested that the expression of TLR7 may be a predictor for rheumatoid arthritis (RA) disease activity and anti-TNFα responsiveness, and targeting TLR7 may suppress chronic progression of RA [23]. Mar et al. indicated that the expression level of TLR-7 was higher in OA fibroblast-like synoviocytes [24]. Due to TLR7 was found to be up-regulated in OA in our study, we speculated that TLR7 may involve in OA. However, down-regulated TLR7 was found in the results of qRT-PCR and western blot, its exact role in OA need to be confirmed in the further study.

CTSS (cathepsin S), encoded by CTSS, is a member of the peptidase C1 family [25]. In addition, CTSS is a lysosomal cysteine proteinase that may participate in the degradation of antigenic proteins to peptides for presentation on MHC class II molecules [25]. Appleton et al. identified increased expression of CTSS in the OA model [26]. The study of Lambert et al. showed that CTSS was significantly up-regulated in inflamed synovial biopsy samples which was confirmed at the protein level by using immunohistochemistry, and it was also significantly up-regulated in cartilage catabolism pathway based on their results [27]. In agreement with previous studies, we observed that CTSS was up-regulated in patients with OA which may imply the importance of CTSS in OA.

TIMP3 (full name, TIMP metallopeptidase inhibitor 3), is a member of TIMP family which is inhibitors of the matrix metalloproteinases, a group of peptidases implicated in degradation of the extracellular matrix (ECM) [28]. In bone, TIMP-3 plays the role as a local cytokine, regulating bone metabolism through suppressing osteoblast differentiation and inducing osteoblast apoptosis [29, 30]. TIMP3 was reported to be associated with RA and arthritis based on a search of the U. S. National Library of Medicine database (MEDLINE) (http://www.ncbi.nlm.nih.gov/IEB/Research/Acembly/av.cgi) [31]. A case-control study in a Chinese Han population linked TIMP3 polymorphism with severe knee OA [32]. In this study, TIMP3 was detected to be dysregulated in patients with OA. Hence, we hypothesized that TIMP3 was involved with OA.

Hsa-miR-17-92 polycistron encodes a cluster of seven miRNAs derived from the c-myc regulated c13orf25 locus at chromosome 13q31.3, including miR-17-5p [33]. MiR-17-5p was reported to have a role as a tumor suppressor in breast cancer cells and as a key regulator of the G1/S phase cell cycle transition [7, 34]. MiR-17-5p may act as an oncogene or a tumor suppressor in different cellular contexts [7]. Although no report linked hsa-miR-17-5p with OA, it was a down-regulated miRNA that covered most DEmRNAs in this study. In addition, TLR7, CTSS and TIMP3 were detected to be target genes of hsa-miR-17-5p. Given the results of our analysis, we hypothesized that hsa-miR-17-5p may participate in OA by regulating TLR7, CTSS and TIMP3.

MiR-20b-5p is transcribed from the miR-106a~ 363 clusters which is reported to be involved in several process [35]. Ma et al. suggested that miR-20b-5p may play a vital role in multiple sclerosis (MS) pathogenesis [36]. In the study of Luo et al., miR-20b-5p was found to play role in promoting myoblast differentiation and repressing myoblast proliferation [35]. Interestingly, hsa-miR-20b-5p was down-regulated in patients with OA. In addition, TLR7 and CTSS were targeted by hsa-miR-20b-5p which indicated that miR-20b-5p may implicate in OA. To our best knowledge, we are the first to report the role of hsa-miR-20b-5p in OA.

## Conclusions

In conclusion, we identified 1068 DEmRNAs (516 up-regulated and 552 down-regulated DEmRNAs), 21 DEmiRNAs (6 up-regulated and 15 down-regulated DEmiRNAs) and 395 DEmiRNA-DEmRNA pairs in synovial tissues of patients with OA, and emphasized the importance of several mRNAs and miRNAs which may implicate in OA. These findings may provide new avenue to understand the mechanism of OA. A limitation of present study is the small sample size for RNA sequencing. The exact function of these mRNAs and miRNAs in OA need to be determined with further large sample research.

## Supplementary information

**Additional file 1 **: **Figure S1** Flow chart of the analyses.

## Data Availability

The raw-data have been uploaded to Gene Expression Omnibus (GEO) (GSE143514, https://www.ncbi.nlm.nih.gov/geo/query/acc.cgi?acc=GSE143514).

## Abbreviations

CTSS: Cathepsin S
DEmiRNA: Differentially expressed miRNA
DEmRNA: Differentially expressed mRNA
ECM: Extracellular matrix
EDTA: Ethylenediaminetetraacetic acid
FPKM: Per million fragments mapped
GO: Gene Ontology
KEGG: Kyoto Encyclopedia of Genes and Genomes
OA: Osteoarthritis
PAMPs: Pathogen-associated molecular patterns
PCR: Polymerase chain reaction
PPI: Protein-protein interaction network
RA: Rheumatoid arthritis
RIN: RNA integrity number
ssRNA: Single-stranded RNA
TIMP3: TIMP metallopeptidase inhibitor 3
TLR7: Toll like receptor 7
