# Supplementary figures and images for: Identification of differentially expressed miRNAs and mRNAs in synovial of osteoarthritis via RNA-sequencing
Source: BMC Med Genet. 2020 Mar 2;21:46. doi: 10.1186/s12881-020-0978-5 (PMC7053084; doi:10.1186/s12881-020-0978-5)

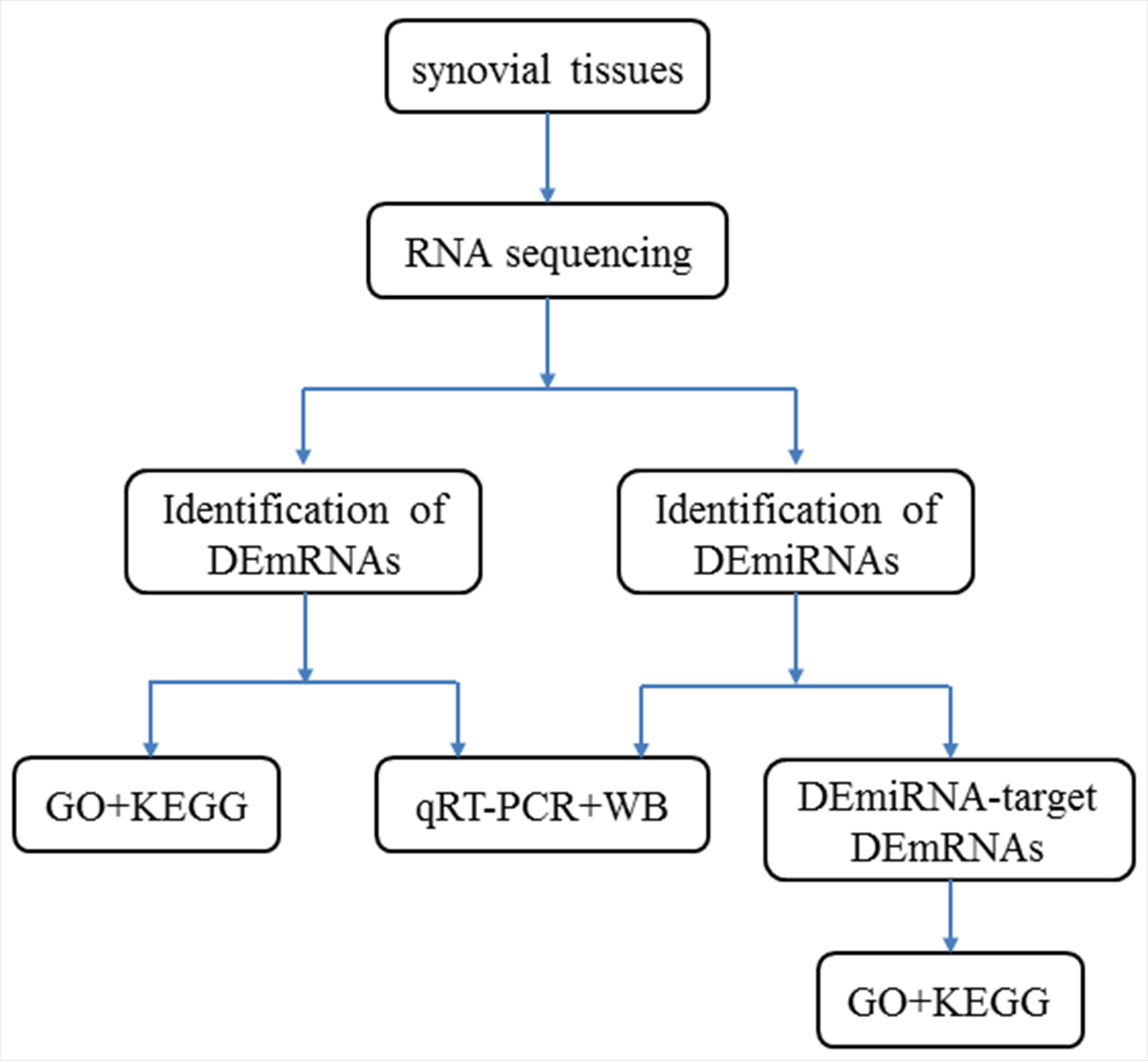

Supplement: Supplementary file 1 — Additional file 1 : Figure S1 Flow chart of the analyses. [file 12881_2020_978_MOESM1_ESM.tif]
